# Effects of probiotics on the prevention and treatment of children with allergic rhinitis: a meta-analysis of randomized controlled trials

**DOI:** 10.3389/fped.2024.1352879

**Published:** 2024-10-03

**Authors:** Xinyi Luo, Huan Wang, Huixia Liu, Yue Chen, Li Tian, Qing Ji, Dengpiao Xie

**Affiliations:** ^1^ The First Affiliated Hospital of Chengdu Medical College Clinical Medical College, Chengdu, Sichuan, China; ^2^ Hospital of Chengdu University of Traditional Chinese Medicine, Chengdu, Sichuan, China; ^3^ Department of Otolaryngology, Chengdu First People’s Hospital, Chengdu, Sichuan, China

**Keywords:** meta-analysis, pediatric allergy rhinitis, probiotics, prevention, treatment

## Abstract

**Background and aim:**

Recent studies have demonstrated the anti-allergic effects of probiotics in humans. However, their role in preventing and treating pediatric allergic rhinitis has not been thoroughly investigated. This study aimed to systematically review the efficacy and preventive effects of probiotics on pediatric allergic rhinitis.

**Methods:**

We systematically searched PubMed, Embase, the Cochrane Central Register of Controlled Trials, and Web of Science databases for all relevant studies on probiotics and pediatric allergic rhinitis. Studies meeting the inclusion criteria were included, data were extracted, and meta-analyses were performed.

**Results:**

A total of 28 studies with 4,765 participants were included in this study. The pooled results showed that the use of probiotics was associated with a significant improvement in total nose symptom scores (SMD, −2.27; 95% CI, −3.26 to −1.29; *P* < 0.00001), itchy nose scores (SMD, −0.44; 95% CI, −0.80 to −0.07; *P* = 0.02), sneezing scores (SMD, −0.47; 95% CI, −0.84 to −0.10; *P* = 0.01), eye symptoms (SMD, −3.77; 95% CI, −5.47 to −2.07; *P* < 0.00001), and Pediatric Rhinoconjunctivitis Quality of Life Questionnaire (SMD, −2.52; 95% CI, −4.12 to −0.92; *P* < 00001). However, the use of probiotics was not associated with the incidence of allergic rhinitis (RR, 0.9; 95% CI, 0.74–1.08; *P* = 0.26).

**Conclusions:**

The present study demonstrated that probiotics were effective and safe for improving pediatric allergic rhinitis symptoms and quality of life. However, probiotics could not prevent pediatric allergic rhinitis.

## 1 Introduction

Allergic rhinitis (AR) is a common disease in children, characterized by nasal congestion, nasal itching, sneezing, and rhinorrhea (1). The prevalence of AR in children continues to increase (2). Based on the International Study of Asthma and Allergies in Childhood, involving 98 countries, up to 45% of children have symptoms of AR (3). About 90% of children with AR symptoms continue to experience them into adulthood (4). Although AR is not life-threatening, it can severely impact the quality of life in children and is associated with sleep-disordered breathing, learning impairment, activity limitations, and emotional disturbances (5). The current management options include allergen avoidance, antihistamines, and intranasal corticosteroids. However, achieving complete symptom resolution of AR is challenging. A survey showed that a large number of patients were dissatisfied with their medication, and up to 60% of patients were interested in finding new allergy treatments (6).

Studies have revealed that gut dysbiosis is associated with allergic diseases such as asthma, eczema, and food allergies (7–9). The allergic diseases are correlated with reduced microbial diversity before the onset of clinical symptoms, further proving the critical role of gut microbiota in these conditions (7, 8). Therefore, probiotic supplementation is considered potentially beneficial in preventing or alleviating allergic diseases. Probiotics are live microorganisms that offer immunological protection to the host by regulating, stimulating, and modulating immune responses. Some meta-analyses have demonstrated the preventive and therapeutic effects of probiotics in children with allergic diseases, such as atopic dermatitis and eczema (9, 10). However, other meta-analyses have failed to prove their preventive effect in developing asthma or wheezing in children (11). No conclusive evidence exists regarding the effects of probiotics on children with AR. The present study aimed to include more high-quality trials to evaluate the role of probiotics in the prevention and treatment of children with AR.

## 2 Materials and methods

The present meta-analysis was conducted and reported in accordance with the Preferred Reporting Items for Systematic Reviews and Meta-analyses guidelines (12). The clinical trials were searched in the following databases: Medline, Embase, the Cochrane Central Register of Controlled Trials, and Web of Science, with a deadline of August 2022. The following keywords were used: “rhinitis, allergic,” “allergic rhinitis,” “allergic rhinitides, seasonal,” “pollen allergy,” “pollinosis,” “probiotics,” “prebiotics,” “children,” “childhood,” “infant,” “teenagers,” “adolescents,” “randomized,” and “trial.” The search was limited to studies in English. In addition, the references of studies or reviews on similar topics were also reviewed to avoid missing potentially relevant studies.

### 2.1 Inclusion and exclusion criteria

The inclusion criteria for preventive studies of AR in children were as follows: (1) infants born in families with a history of allergic disease, infants with food allergies considered at high risk of developing atopy, or healthy children, (2) in the probiotic group, the mother during pregnancy and/or the infant took probiotics, (3) in the control group, the participants received the same therapy except for probiotics, and (4) the outcome reported the incidence of AR.

The inclusion criteria for treatment studies of AR in children were as follows: (1) children diagnosed with AR, based on clinical examination, skin-prick tests, and serum allergen-specific immunoglobulin E (IgE) (2) in the probiotic group, children took probiotics, including all types of probiotic strains, (3) in the control group, children received the same therapy as the probiotic group, except for probiotics, and (4) the primary outcome included nose symptoms of AR and the secondary outcome included eye symptoms, Pediatric Rhinoconjunctivitis Quality of Life Questionnaire (PRQLQ), and immunological parameters.

The exclusion criteria were as follows: (1) non-human studies; (2) non-comparative studies; (3) non-randomized controlled trials (RCTs); (4) full text not available; (5) data used in more than one study; in such cases, we included only one study and excluded the others; (6) repeatedly published trials; (7) case reports, comments, letters, reviews, and retrospective studies; (8) ongoing trials without results; and (9) no relevant outcomes.

### 2.2 Study selection, data extraction, and quality assessment

Two independent investigators assessed the titles abstracts, and full-text articles based on the inclusion and exclusion criteria. Any disagreements were resolved through discussion or by consulting a third investigator. Two investigators independently extracted data from each eligible study, including the name of the first author, year of publication, study design, the regimen of intervention in the probiotic group (including the probiotic dose, strain of probiotics, and treatment course), study duration, outcomes, and adverse events. When a study compared more than one probiotic group with one control group, the number of participants in the control group was divided by the number of probiotic groups. When the outcomes were reported at different time points, the data were extracted from the last time point. The study quality was assessed using the Cochrane risk-of-bias tool, which included selection bias, performance and detection bias, attrition bias, reporting bias, and other sources of bias. Two independent investigators performed the assessment, and any discrepancies were resolved by a third author.

### 2.3 Data synthesis and analysis

Odds ratio (OR) was used to assess the incidence of AR. Weighted mean difference (WMD) or standardized mean difference (SMD) was used to assess the AR symptoms and cytokines. When outcomes of the included studies were reported using different measurement scales, SMD was used to assess the pooled effect. The fixed-effects model was used to assess the pooled effect when low heterogeneity was considered; otherwise, the random-effects model was used. The heterogeneity among studies was assessed using the inconsistency index (*I*^2^). We considered *I*^2^ ≤ 25% as low heterogeneity, between 25% and 50% as moderate heterogeneity, and >50% as significant heterogeneity (13).

### 2.4 Publication bias and sensitivity analysis

Potential publication bias was assessed using a funnel plot. The sensitivity analysis was performed by deleting one study at a time to assess the stability of the pooled results. The data were analyzed using Review Manager, version 5.3 (Oxford, UK) or Stata 15.

## 3 Results

### 3.1 Literature selection and study characteristics

**Figure 1:**
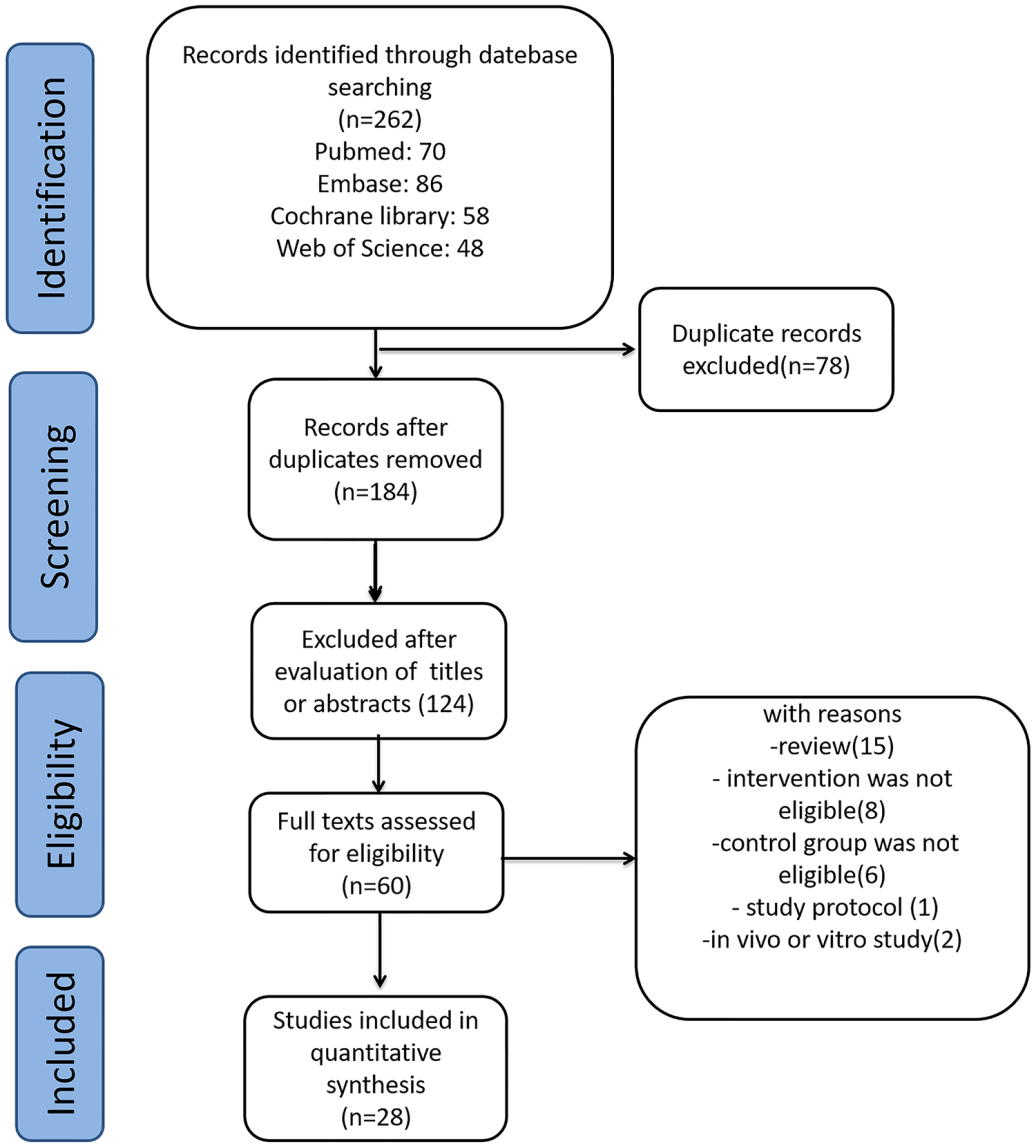
Selection of studies for the meta-analysis review.

We identified 262 relevant publications from the databases. After removing 78 duplicate publications using Endnote, 184 studies were excluded based on titles and abstracts. Further, 156 studies were excluded based on the inclusion and exclusion criteria. Finally, 28 studies met the criteria and were included in this meta-analysis. The other studies were excluded for reasons such as being reviews, *in vivo* studies, study protocols, or having ineligible intervention or control groups. A flow diagram depicting the selection of studies is shown in Figure 1.

The characteristics of the included studies are presented in Table 1. A total of 28 trials with 4,765 participants were included in this systematic review and meta-analysis (17–44). Among these, 14 trials assessed the preventive effects of probiotics (17, 18, 20, 23, 24, 26, 27, 29, 30, 37–39, 42, 43), whereas the other 14 trials assessed the treatment effects of probiotics (19, 21, 22, 25, 28, 31–36, 40, 41, 44). In the studies assessing the preventive effects of probiotics, seven trials included pregnant mothers and their children (14, 15, 21, 23, 26, 27, 40). Probiotics included *Bifidobacterium*, *Lactobacillus*, *Enterococcus*, *Escherichia*, and *Clostridium butyricum* strains. The treatment duration of probiotics ranged from 3 weeks to 39 months.

**Table 1:** Basic characteristics of subjects and treatments of trials.

Reference	Type	Country	Sample size	Intervention	Control	Study duration	Adverse
Abrahamsson et al. (14)	RCT	Sweden	188	Mother from 36 weeks gestation took L rreuteri (1 × 108 CFUs, daily) until delivery. After birth, the baby continued the same product as the mother up to 12 months of age.	Placebo	2 years	Spitting-up, colic, and constipation
Allen et al. (15)	RCT	UK	391	Mother from 36 weeks gestation and their infants to age 6 months received (Lactobacillus salivarius CUL61, Lactobacillus paracasei CUL08, Bifidobacterium animalis subspecies lactis CUL34 and Bifidobacterium bifidum CUL20; total 1,010 CFUs/day).	Placebo	2 years	Not reported
Anania et al. (16)	RCT	Italy	203	Children received conventional therapy (local corticosteroids and/or oral antihistamines) and a probiotic mixture of 2 × 10^9^ CFUs of Bifidobacterium animalis subsp. Lactis BB12 and 2 × 10^9^ of enterococcus faecium L3 strain daily for 3 months.	Local corticosteroids and/or oral antihistamines	3 months	Not reported
Berni Canani et at. (17)	RCT	Italy	192	Children received hydrolysed casein formula containing the probiotic LGG daily for 36 months.	Hydrolysed casein formula	36 months	No adverse event
Chen et at. (18)	RCT	China	105	Children received 1 capsule of L.gasseri (2 × 10^9^ CFUs/capsule) twice a day for 8 weeks.	placebo	10 weeks	No adverse event
Ciprandi et al. (19)	RCT	Italy	20	Children received Bacillus clausii (2 billion spores/vial) three vials a day plus levocetirizine for 3 weeks.	Levocetirizine	3 weeks	Not reported
Corsello et al. (20)	RCT	Italy	126	Children received daily 7 grams of cow's skim milk fermented with L. paracasei daily for 3 months.	Placebo	3 months	No adverse event
Dotterud et al. (21)	RCT	Norway	278	Women received probiotic milk (containing 5 × 10^10^ CFUs of LGG, Bifidobacterium animalis subsp. Lactis Bb-12, and 5 × 10^9^ of L. acidophilus La-5) from 36 weeks of gestation to 3 months postnatally during breastfeeding.	Placebo	2 years	No adverse event
Giovannini et al. (22)	RCT	Italy	116	Children received fermented milk containing Lactobacillus bulgaricus (10^7^ CFU/ml), Streptococcus thermophiles (10^8^ CFU/ml), Lactobacillus casei (10^8^ CFU/ml) daily for 12 months.	Placebo	12 months	Not reported
Gorissen et al. (23)	RCT	Netherlands	83	Women received probiotic mixture consisting of Bifidobacterium bifidum, Bifidobacterium lactis and Lactococcus lactis during the pregnancy, and baby continued the same product for the first year of life.	Placebo	6 years	Not reported
Jensen et al. (24)	RCT	Australia	108	Children received 3 × 10^9^ Lactobacillus acidophilus daily for daily for 6 months.	Placebo	5 years	Not reported
Jerzynska et al. (25)	RCT	USA	46	Children received sublingual immunotherapy plus LGG (3 × 10^10^/dose) once daily for 5 months.	Sublingual immunotherapy	5 months	Not adverse events
Kalliomäki et al. (26)	RCT	Finland	107	Mother received 1 × 10^10^ CFUs of LGG daily for 4 weeks before expected delivery. After delivery, either the mother or the infant consumed the probiotics for 6 months.	Placebo	2 years	Not reported
Kuitunen et al. (27)	RCT	Finland	891	From 36 weeks of gestation, mothers took 5 × 10^9^ CUF Lactobacillus rhamnosus GG, 5 × 10^9^ CUF L rhamnosus LC705, 2 × 10^9^ Bifidobacterium breve Bb99, and 2 × 10^9^ CUF Propionibacterium freudenreichii ssp. shermanii JS twice daily. Their infants received the same probiotics once daily during the 6 months from birth.	Placebo	5 years	1 adverse event in probiotic group
Lin et al. (28)	RCT	China	199	Children received 4 × 10^9^ CFUs/g of Lactobacillus salivarius daily for 12 weeks.	Placebo	12 weeks	Not reported
Lin et al. (29)	RCT	China	60	Children received Lactobacillus paracasei (5 × 10^9^ CUFs/ capsule) daily plus levocetirizine for 8 weeks.	Levocetirizine plus placebo	12 weeks	No serious adverse events
Lue et al. (30)	RCT, and crossover	China	57	Children took Lactobacillus johnsonii EM1 (1 × 10^10^ CFU/capsule) plus levocetirizine (5 mg) daily for 12 weeks.	Levocetirizine	12 weeks	No serious adverse events
Miraglia et al. (31)	RCT	Italy	40	Children took Bifidobacteria mixture, B longum BB536 (3 × 10^9^ CFU), B infantis M-63 (1 × 10^9^ CFU), and B breve M-16V (1 × 10^9^ CFU) daily for 4 weeks.	Placebo	4 weeks	Not reported
Ouwehand et al. (32)	RCT	Finland	41	Children took a 5 × 10^9^ CFU of a combination of 25% Lactobacillus acidophilus and 75% Bifidobacterium lactis daily for 3 months.	Placebo	3 months (birch pollen season)	Not reported
Peng et al. (33)	RCT	China	90	Children took 5 × 10^9^ CFU heat-killed or live L. paracasei daily for 30 days.	Placebo	30 days	Not reported
Roßberg and Keller (34)	RCT	Germany	402	Children took heat-killed escherichia coli and enterococcus faecalis three times daily from 5 weeks until 7 months of life.	Placebo	6–11 years	Not reported
Scalabrin et al. (35)	RCT	USA	68	Children took extensively hydrolyzed casein formula with 10^6^ CFU LGG from 14 to 120 days of age.	Extensively hydrolyzed casein formula	5 years	No serious adverse events
Schmidt et al. (36)	RCT	Denmark	260	Children took 1 × 10^9^ CFU of a combination of LGG and bifidobacterium animalis subsp lactis for 6 months	Placebo	6 months	Not reported
Sumadiono et al. (37)	RCT	Japan	41	Children took a sachet of probiotic (Protexin®) and 10 mg cetirizine for 7 weeks	10 mg cetirizine	7 weeks	Not reported
Wang et al. (38)	RCT	China	80	Children took 2 × 10^9^ CFU Lactobacillus paracasei-33 daily for 30 days	Placebo	30 days	No serious adverse events
West et al. (39)	RCT	Sweden	117	Children took Lactobacillus paracasei ssp paracasei F19 (10^8^ CUF) from 4 to 13 months of age.	Placebo	8–9 years	Not reported
Wickens et al. (40)	RCT	Australia	298	Pregnant women with 35-week gestation took either L. rhamnosus HN001 (6 × 10^9^ colony-form- ing units/d) or B. lactis HN019 (9 × 10^9^ colony-forming units/d). Women continued taking the study capsules till 6 months’ postpartum, or the end of breastfeeding. The infants took same probiotics daily from birth till age 2 year.	Placebo	11 years	Not reported
Xu et al. (41)	RCT	China	158	Children took two capsules of clostridium butyricum (420 mg/capsule) or plus allergen specific immunotherapy twice daily for 6 months	Either placebo or allergen specific immunotherapy	12 months	Not reported

CFUs, colony forming units; LGG, L. rhamnosus GG.

### 3.2 Risk of bias

**Figure 2:**
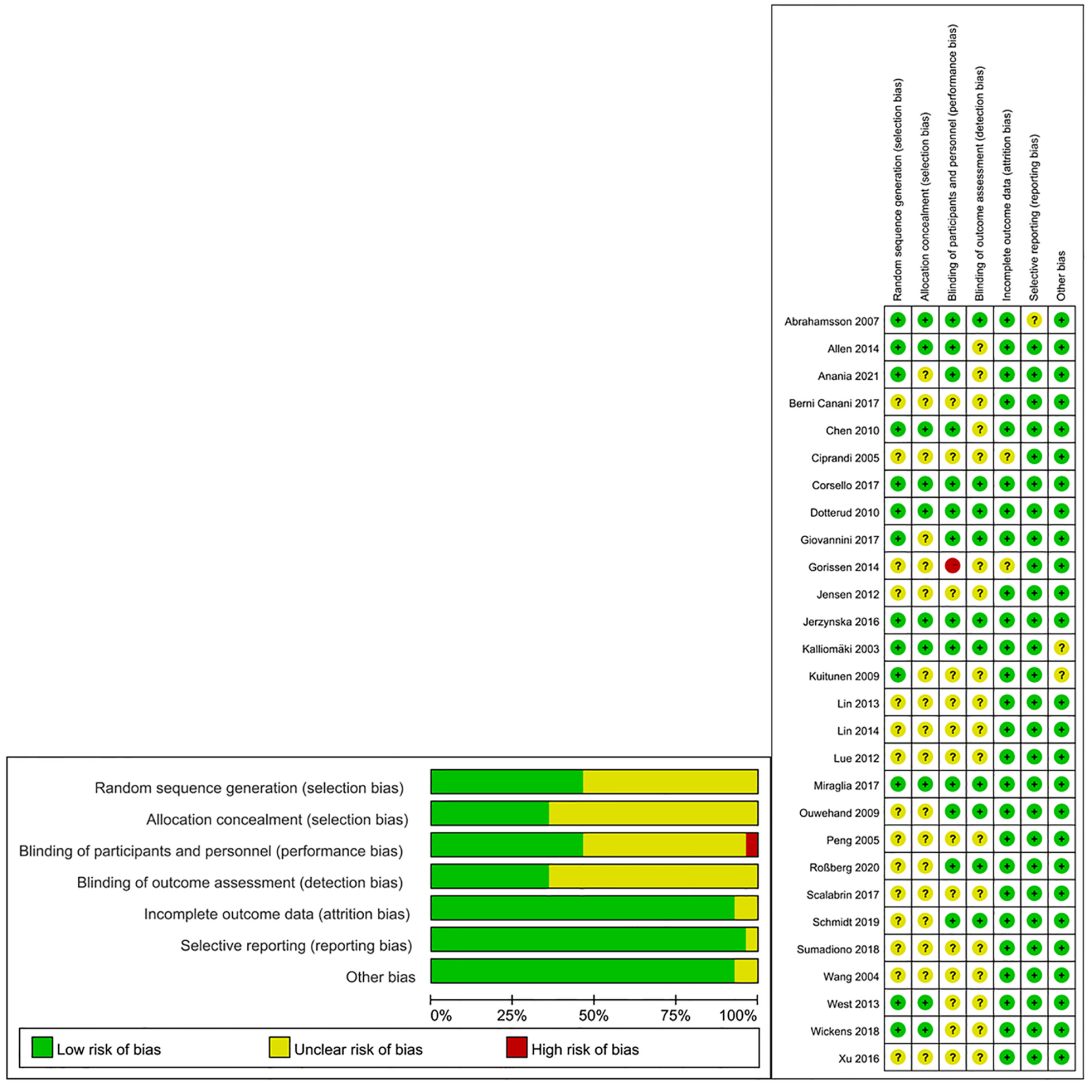
Summary of risk of bias of included studies.

The summary of the risk of bias in the present meta-analysis is shown in Figure 2. All studies reported that they were randomized trials, with concrete methods of randomization reported in several studies (17, 18, 19, 21, 23–25, 28, 31, 34). However, insufficient information was available to judge the masking method as “low risk” or “high risk” for most of the studies. One trial was a single-blinded study and was considered “high risk” for performance bias (23).

### 3.3 Probiotics for preventing allergic diseases

**Figure 3:**
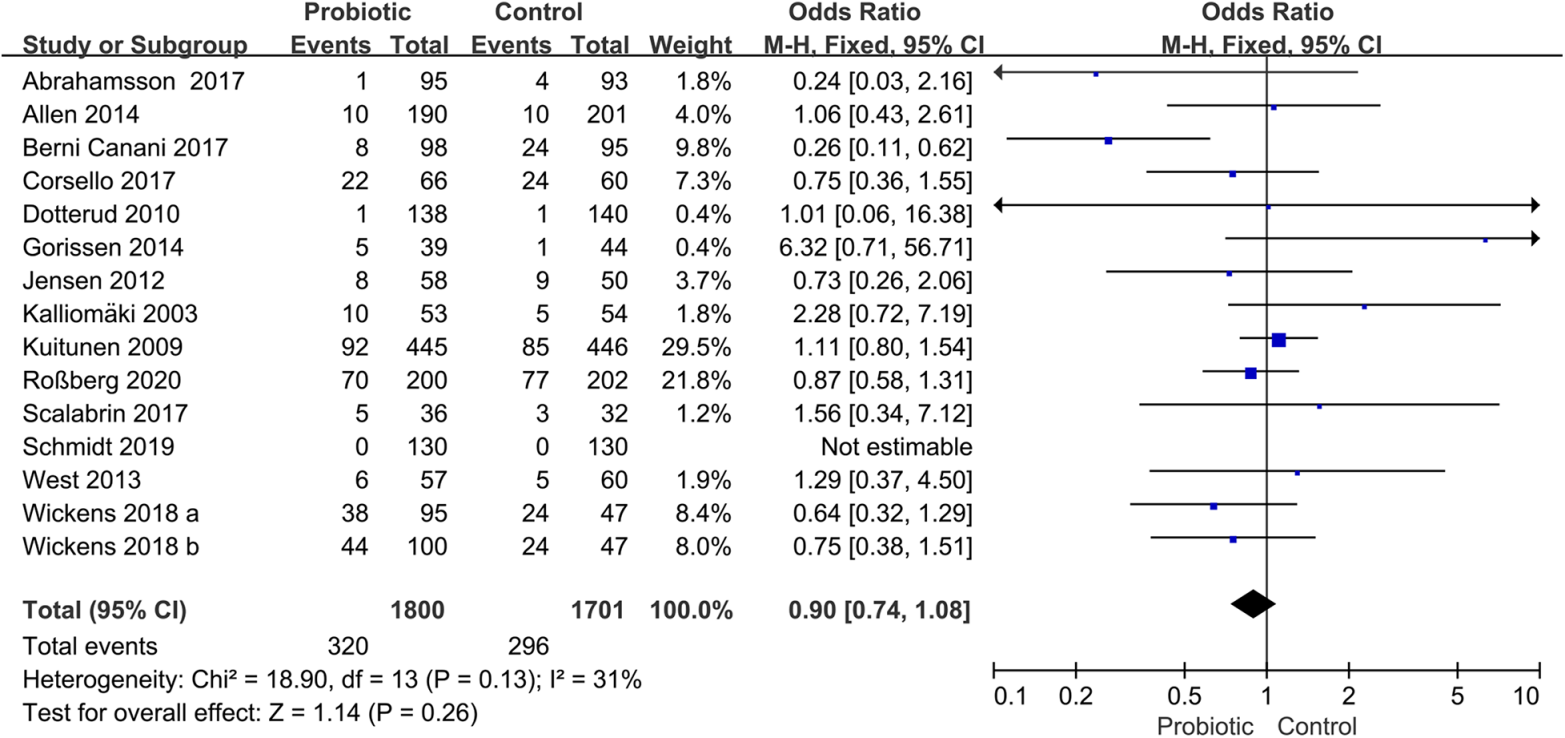
Forest plot for the effects of probiotics on the incidence of children with AR. AR, Allergic rhinitis.

Fourteen trials assessed the preventive role of probiotics for children with AR (17, 18, 20, 23, 24, 26, 27, 29, 30, 37–39, 42, 43). The pooled effect of the meta-analysis showed that the use of probiotics was not associated with the incidence of AR (OR, 0.90; 95% CI, 0.74–1.08; *P* = 0.26; *I*^2^ = 31%) (Figure 3). The subgroup analysis based on pregnant mothers taking probiotics (pregnant mother group) or only children taking probiotics (children group) revealed no significant difference compared with the control group within either the pregnant mother group (OR, 1.02; 95% CI, 0.80–1.31; *P* = 0.86; *I*^2^ = 21%) or the children group (OR, 0.75; 95% CI, 0.56–1.00; *P* = 0.05; *I*^2^ = 37%) (Supplementary Figure S1). Another subgroup analysis based on children with high risk or non-high-risk of allergy showed no significant differences in the incidence of AR between the probiotic and control groups in children with high risk (OR, 0.89; 95% CI, 0.73–1.09; *P* = 0.26; *I*^2^ = 50%) or non-high risk (OR, 0.95; 95% CI, 0.54–1.66; *P* = 0.84; *I*^2^ = 0%) (Supplementary Figure S2).

### 3.4 Probiotics for symptom scores of AR

**Figure 4:**
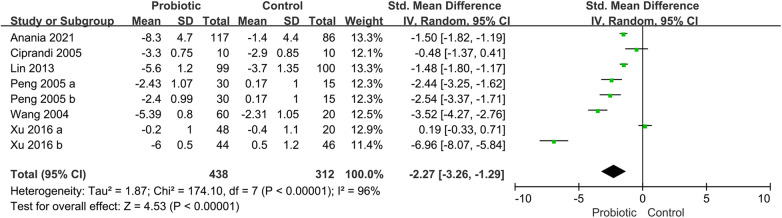
Forest plot for the effects of probiotics on TSS in children with AR. AR, Allergic rhinitis; TSS, total symptom score.

Six trials reported the total symptom score (TSS), including assessments of nasal congestion, sneezing, nasal itching, and rhinorrhea (16, 19, 28, 33, 38, 41). The pooled results showed that the use of probiotics was associated with a significant improvement in TSS (SMD, −2.27; 95% CI, −3.26 to −1.29; *P* < 0.00001; *I*^2^ = 96%) (Figure 4). The subgroup analysis based on children receiving probiotics and other therapies such as corticosteroids or antihistamines (combination group) or only probiotics intervention (monotherapy group). The subgroup analysis revealed no improvement in TSS in the combination group (SMD, −2.94; 95% CI, −5.90 to 0.01; *P* = 0.05; *I*^2^ = 98%). Another subgroup analysis revealed no significant improvement in the probiotic group compared with the placebo group (SMD, −1.93; 95% CI, −3.09 to −0.76; *P* = 0.001; *I*^2^ = 95%) (Supplementary Figure S3).

Two trials reported the scores of itchy nose and sneezing (29, 30). The pooled results showed that the use of probiotic was associated with a significant improvement of itchy nose scores (SMD, −0.44; CI, −0.80, −0.07; *P* = 0.02; I^2^ = 0%), Supplementary Figure S4, and sneezing scores (SMD, −0.47; CI, −0.84, −0.10; *P* = 0.01; I^2^ = 41%), Supplementary Figure S5.

Three trials reported the eye symptoms (28, 33, 38). The pooled results showed that the use of probiotic was associated with a significant improvement of eye symptoms (SMD, −3.77; CI, −5.47, −2.07; *P* < 0.00001), with significant heterogeneity (I^2^ = 95%), Supplementary Figure S6.

### 3.5 Probiotics for PRQLQ

Five trials reported the PRQLQ (32–34, 36, 41), including nasal symptoms, ocular symptoms, practical problems, activity limitations, and other symptoms (42). The pooled results showed that the use of probiotics was associated with a significant improvement in PRQLQ (SMD, −2.52; 95% CI, −4.12 to −0.92; *P* < 0.00001, *I*^2^ = 96%) (Supplementary Figure S7).

### 3.6 Probiotics for immunological parameters

Five trials reported the effects of probiotics on IgE (18, 22, 28, 30, 41). The pooled results showed no significant difference between the two groups (SMD, −0.77; 95% CI, −1.53 to −0.01; *P* = 0.05; *I*^2^ = 95%) (Supplementary Figure S8). Four trials reported the effects of probiotics on interleukin (IL)-10 levels. The pooled results showed no significant difference between the two groups (SMD, −0.15; 95% CI, −0.43 to 0.12; *P* = 0.28; *I*^2^ = 0%) (Supplementary Figure S9).

### 3.7 Adverse events

Sixteen trials did not report adverse events during the study (18, 19, 25–27, 29, 31, 34–37, 39, 40, 42–44). Five trials reported no adverse events in the probiotic and control groups (17, 18, 20, 21, 25). Four trials reported no serious adverse events in the probiotic and control groups (29, 30, 35, 38). One trial reported one adverse event in the probiotic group (27). One trial reported mild adverse events, including spitting up, abdominal colic, and constipation, with no significant difference between the two groups (14).

### 3.8 Publication bias and sensitivity analysis

The funnel plot analysis showed symmetry for the events of AR in children (Supplementary Figure S10). Similarly, Egger's test did not detect a significant publication bias (*P* > 0.05). The sensitivity analysis was assessed by leave-one-out analysis for events of AR in children, and the pooled results and heterogeneity did not significantly change.

## 4 Discussion

The present meta-analysis included 28 trials with 4,765 participants. The results revealed that probiotic supplementation alleviated the symptoms of AR and improved the PRQLQ in children.

However, it could not prevent the development of AR in children and had no significant impact on regulating IgE and IL-10 levels in children with AR. Additionally, probiotics were shown to be safe and not associated with an increased risk of side effects.

Probiotics have been widely explored for preventing allergic diseases, and evidence has been established for their supplementation in reducing the development of certain allergic diseases. Children who received *Lactobacillus* or *Bifidobacterium* supplementation were associated with a reduced prevalence of eczema and wheezing (40, 43, 44). However, whether probiotics can effectively prevent AR remains unclear; some studies have even demonstrated that probiotics may increase the incidence of AR (45). The present study provided reliable evidence that probiotics were not associated with increasing or reducing the incidence of AR. Interestingly, both atopic eczema and AR are allergic diseases, and AR often co-occurs with eczema, suggesting a shared pathogenesis or mechanism (46). However, probiotics have a different impact on the incidence of eczema and AR in children, and the underlying mechanisms remain unclear. Another aspect to consider is the development of gut microbes in infants. The transmission of maternal microbes during delivery plays a vital role in colonizing the infant's gut (47). Some studies even suggest that the existence of bacteria in infants begins prenatally (48), indicating that microbes may influence the immune system before birth. However, no evidence shows the role of probiotics in preventing allergic diseases in adults, indicating that earlier probiotic supplementation might have a better effect in preventing AR. Therefore, the beginning of probiotic supplementation in pregnant women or infants might have a different effect on AR. However, the pooled results showed that the initiation of probiotic supplementation by either pregnant women or infants was not associated with the incidence of AR. Finally, we found that probiotic supplementation was not associated with the incidence of AR in children with a high or non-high risk for allergy.

Regarding the effects of probiotics on children with AR, most of the included studies showed that probiotics improved the severity of AR symptoms; two studies found that probiotics reduced the occurrence of AR symptoms (18, 37). The mechanism of probiotics in improving the symptoms of AR has not yet been completely explained, but some possible mechanisms have been suggested. It is suggested that the regulation of T helper (Th) cells may be involved in the protective effect of probiotics. The cells are classified into two subsets, Th1 and Th2, and maintaining the balance of Th1/Th2 cells is crucial for regulating the adaptive immune response. A Th2-dominant condition has been shown to increase the risk of allergic diseases. Probiotics have been found to promote the function of Th1 cells while inhibiting Th2 responses, which helps control the overproduction of IgE and pro-inflammatory cytokines (49, 50). In addition, other evidence suggests that probiotics increase the number of regulatory T cells by changing the composition of intestinal microflora and modifying antigen-specific serum IgE levels in animal models (51). Probiotics have been shown to improve the barrier function of the intestinal mucosa, reducing the leakage of antigens through the mucosa and improving the local immune system by enhancing the immunoglobulin A response (52, 53). However, further studies are needed to explore how probiotics can improve AR symptoms but not prevent the development of AR in children.

Based on the results of this study, we do not recommend probiotic supplementation for preventing AR in children. However, we recommend probiotic supplementation for alleviating AR symptoms in children. Several advantages of probiotics in alleviating children with AR exist. First, probiotics can be mixed with milk or yogurt, making it easier for children or infants to take. Second, probiotics are generally safe and have few or only mild side effects. Third, probiotics also can improve gastrointestinal dysfunctions, such as diarrhea, constipation, and indigestion. However, probiotics cannot replace anti-AR drugs, such as antihistamines or steroids, during acute episodes of AR.

## 5 Strengths and limitations

This was not the first meta-analysis to assess the effect of probiotics in AR. However, it had several strengths compared with other meta-analyses. First, in our meta-analysis, we studied both the preventive and therapeutic effects of probiotics in AR, whereas other studies only studied the preventive or therapeutic effects of probiotics. Second, we included a large number of trials and participants, which allowed us to obtain more stable results. Third, this meta-analysis included only children as study participants, reducing heterogeneity between children and adults.

This study had several limitations as well. First, high heterogeneity was observed in some pooled results, including TSS and PRQLQ and IgE levels. The heterogeneity might have originated from different probiotic strains, treatment duration, and symptom severity. However, due to the low number of trials, we did not perform subgroup analyses to explore the cause of heterogeneity. Second, we did not explore the effect of different probiotic strains on AR, and this was also due to the low number of trials. Third, our search was restricted to publications in English; hence, some studies in other languages might have been missed.

## 6 Conclusions

The present study demonstrated that probiotics effectively and safely improved pediatric AR symptoms and PRQLQ. However, probiotics could not prevent AR in children.

## Data Availability

The original contributions presented in the study are included in the article/Supplementary Material, further inquiries can be directed to the corresponding authors.
