# Supplementary material for: Effects of probiotics on the prevention and treatment of children with allergic rhinitis: a meta-analysis of randomized controlled trials
Source: Front Pediatr. 2024 Oct 3;12:1352879. doi: 10.3389/fped.2024.1352879 (PMC11484092; doi:10.3389/fped.2024.1352879)
Supplement: Supplementary file 1 [file Datasheet1.pdf]

Supplementary file

Effect of probiotics in prevention and treatment for children with allergic rhinitis: a meta-analysis of randomized controlled trials

Supplementary Table 1. Search strategy in PubMed

PubMed		
1	"Rhinitis, Allergic"[Mesh]	22810
2	((Allergic Rhinitides[Title/Abstract]) OR (Rhinitides, Allergic[Title/Abstract])) OR (Allergic Rhinitis[Title/Abstract])	25733
3	"Child"[Mesh]	2065832
4	(Children[Title/Abstract])	1161218
5	"Pediatrics"[Mesh]	62247
6	"Infant"[Mesh]	1214577
7	(Infants[Title/Abstract])	281839
8	"Infant, Newborn"[Mesh]	651299
9	((((((Infants, Newborn[Title/Abstract]) OR (Newborn Infant[Title/Abstract])) OR (Newborn Infants[Title/Abstract])) OR (Newborns[Title/Abstract])) OR (Newborn[Title/Abstract])) OR (Neonate[Title/Abstract])) OR (Neonates[Title/Abstract])	268913
10	"Adolescent"[Mesh]	2169220
11	((((((((((((((Male Adolescents[Title/Abstract]) OR (Male Adolescent[Title/Abstract])) OR (Adolescent, Male[Title/Abstract])) OR (Adolescents, Male[Title/Abstract])) OR (Female Adolescents[Title/Abstract])) OR (Female Adolescent[Title/Abstract])) OR (Adolescent, Female[Title/Abstract])) OR (Adolescents, Female[Title/Abstract])) OR (Youths[Title/Abstract])) OR (Youth[Title/Abstract])) OR (Adolescents[Title/Abstract])) OR (Adolescence[Title/Abstract])) OR (Teens[Title/Abstract])) OR (Teen[Title/Abstract])) OR (Teenagers[Title/Abstract])) OR (Teenager[Title/Abstract])	341828
12	"Probiotics"[Mesh]	21994
13	(Probiotic[Title/Abstract])	20753
14	Randomized controlled trial OR Controlled clinical trial OR Randomized OR Placebo OR Randomly	1668268
15	(1 OR 2) and (3 OR 4 OR 5 OR 6 OR 7 OR 8 OR 9 OR10 OR 11) and (12 OR 13) and (14)	54

Study or Subgroup	Probiotic Events	Probiotic Total	Control Events	Control Total	Weight	Odds Ratio M-H, Fixed, 95% CI
**1.1.1 Mother**						
Abrahamsson 2007	1	95	4	93	1.8%	0.24 [0.03, 2.16]
Allen 2014	10	190	10	201	4.0%	1.06 [0.43, 2.61]
Dotterud 2010	1	138	1	140	0.4%	1.01 [0.06, 16.38]
Gorissen 2014	5	39	1	44	0.4%	6.32 [0.71, 56.71]
Kalliomäki 2003	10	53	5	54	1.8%	2.28 [0.72, 7.19]
Kuitunen 2009	92	445	85	446	29.5%	1.11 [0.80, 1.54]
Wickens 2018 a	38	95	24	47	8.4%	0.64 [0.32, 1.29]
Wickens 2018 b	44	100	24	47	8.0%	0.75 [0.38, 1.51]
**Subtotal (95% CI)**		**1155**		**1072**	**54.3%**	**1.02 [0.80, 1.31]**
Total events	201		154			
Heterogeneity: Chi^2 = 8.89, df = 7 (P = 0.26); I^2 = 21%						
Test for overall effect: Z = 0.17 (P = 0.86)						
**1.1.2 Children**						
Berni Canani 2017	8	98	24	95	9.8%	0.26 [0.11, 0.62]
Corsello 2017	22	66	24	60	7.3%	0.75 [0.36, 1.55]
Jensen 2012	8	58	9	50	3.7%	0.73 [0.26, 2.06]
Roßberg 2020	70	200	77	202	21.8%	0.87 [0.58, 1.31]
Scalabrin 2017	5	36	3	32	1.2%	1.56 [0.34, 7.12]
Schmidt 2019	0	130	0	130		Not estimable
West 2013	6	57	5	60	1.9%	1.29 [0.37, 4.50]
**Subtotal (95% CI)**		**645**		**629**	**45.7%**	**0.75 [0.56, 1.00]**
Total events	119		142			
Heterogeneity: Chi^2 = 7.91, df = 5 (P = 0.16); I^2 = 37%						
Test for overall effect: Z = 1.96 (P = 0.05)						
**Total (95% CI)**		**1800**		**1701**	**100.0%**	**0.90 [0.74, 1.08]**
Total events	320		296			
Heterogeneity: Chi^2 = 18.90, df = 13 (P = 0.13); I^2 = 31%						
Test for overall effect: Z = 1.14 (P = 0.26)						
Test for subgroup differences: Chi^2 = 2.58, df = 1 (P = 0.11), I^2 = 61.2%						

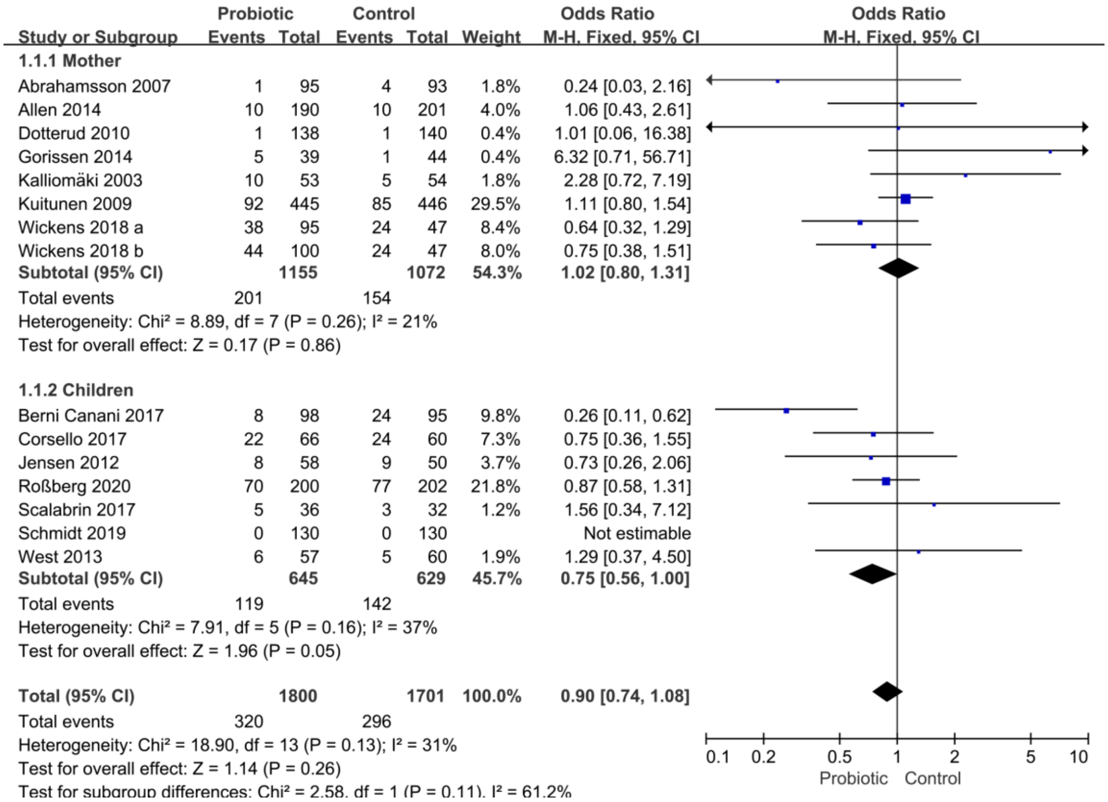

Supplementary Figure. 1 Forest plot for effect of probiotics on incidence of AR in pregnant mother group or children group. AR: allergic rhinitis

Study or Subgroup	Probiotic Events	Probiotic Total	Control Events	Control Total	Weight	Odds Ratio M-H, Fixed, 95% CI
**1.2.1 High risk**						
Abrahamsson 2007	1	95	4	93	1.8%	0.24 [0.03, 2.16]
Allen 2014	10	190	10	201	4.0%	1.06 [0.43, 2.61]
Berni Canani 2017	8	98	24	95	9.8%	0.26 [0.11, 0.62]
Gorissen 2014	5	39	1	44	0.4%	6.32 [0.71, 56.71]
Jensen 2012	8	58	9	50	3.7%	0.73 [0.26, 2.06]
Kalliomäki 2003	10	53	5	54	1.8%	2.28 [0.72, 7.19]
Kuitunen 2009	92	445	85	446	29.5%	1.11 [0.80, 1.54]
Roßberg 2020	70	200	77	202	21.8%	0.87 [0.58, 1.31]
Wickens 2018 a	38	95	24	47	8.4%	0.64 [0.32, 1.29]
Wickens 2018 b	44	100	24	47	8.0%	0.75 [0.38, 1.51]
**Subtotal (95% CI)**		**1373**		**1279**	**89.1%**	**0.89 [0.73, 1.09]**
Total events	286		263			
Heterogeneity: Chi^2 = 17.82, df = 9 (P = 0.04); I^2 = 50%						
Test for overall effect: Z = 1.14 (P = 0.26)						
**1.2.2 Non-high risk**						
Corsello 2017	22	66	24	60	7.3%	0.75 [0.36, 1.55]
Dotterud 2010	1	138	1	140	0.4%	1.01 [0.06, 16.38]
Scalabrin 2017	5	36	3	32	1.2%	1.56 [0.34, 7.12]
Schmidt 2019	0	130	0	130		Not estimable
West 2013	6	57	5	60	1.9%	1.29 [0.37, 4.50]
**Subtotal (95% CI)**		**427**		**422**	**10.9%**	**0.95 [0.54, 1.66]**
Total events	34		33			
Heterogeneity: Chi^2 = 1.05, df = 3 (P = 0.79); I^2 = 0%						
Test for overall effect: Z = 0.20 (P = 0.84)						
**Total (95% CI)**		**1800**		**1701**	**100.0%**	**0.90 [0.74, 1.08]**
Total events	320		296			
Heterogeneity: Chi^2 = 18.90, df = 13 (P = 0.13); I^2 = 31%						
Test for overall effect: Z = 1.14 (P = 0.26)						
Test for subgroup differences: Chi^2 = 0.04, df = 1 (P = 0.85), I^2 = 0%						

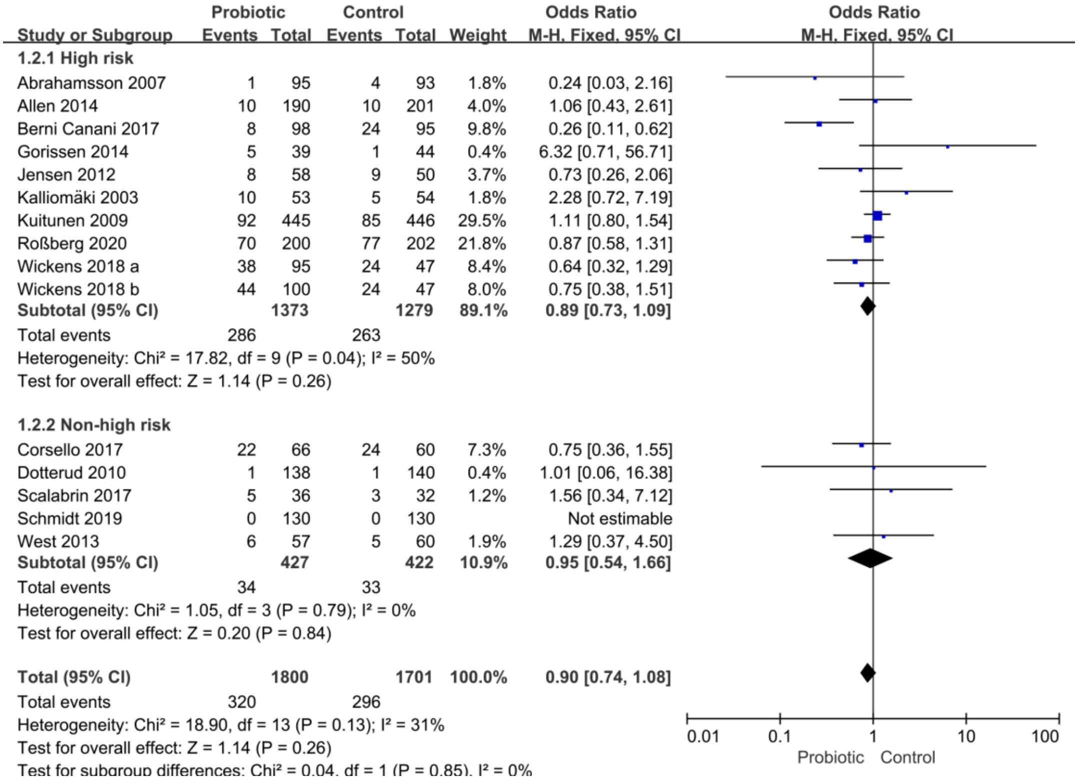

Supplementary Figure. 2 Forest plot for effect of probiotics on incidence of AR in children with high-risk group or non-high risk group. AR: allergic rhinitis

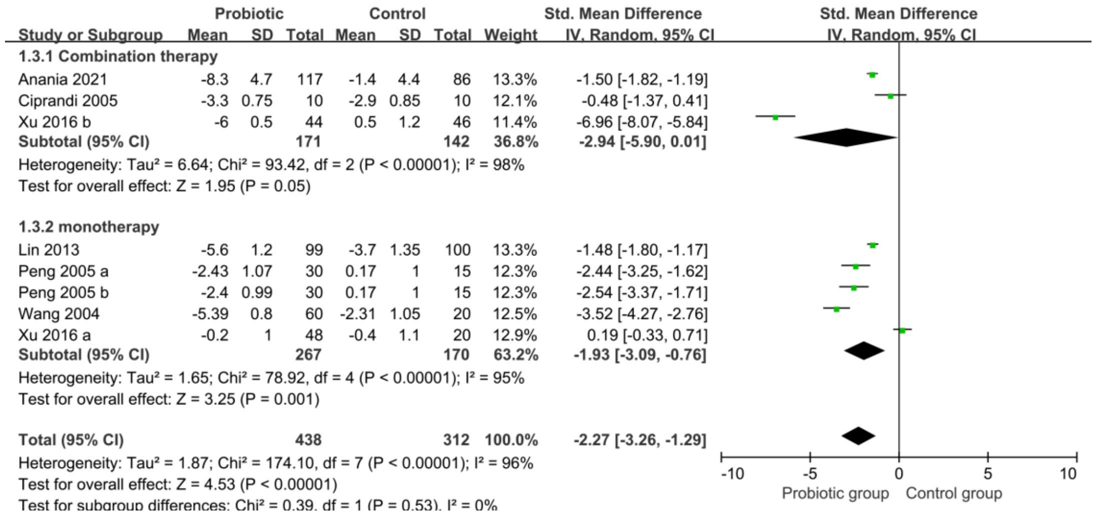

Supplementary Figure. 3 Forest plot for effect of probiotics on TSS in combination therapy group or monotherapy group. TSS: total nose symptoms

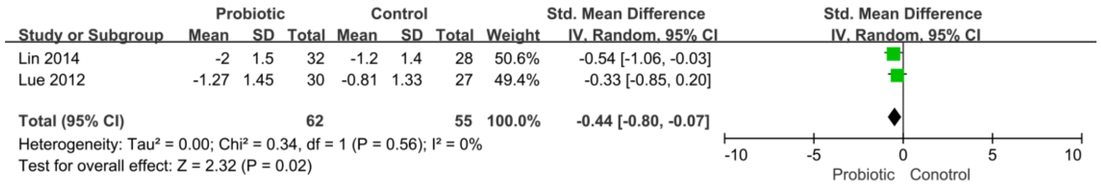

Supplementary Figure. 4 Forest plot for effect of probiotics on itch nose scores.

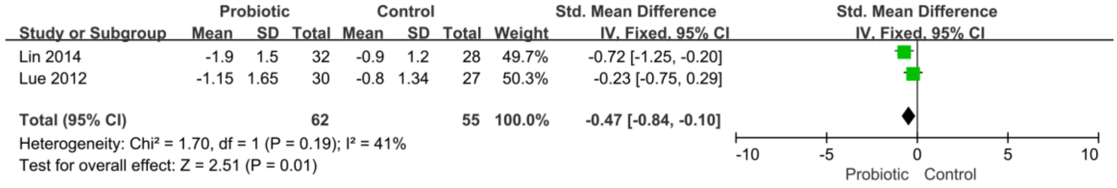

Supplementary Figure. 5 Forest plot for effect of probiotics on sneezing scores.

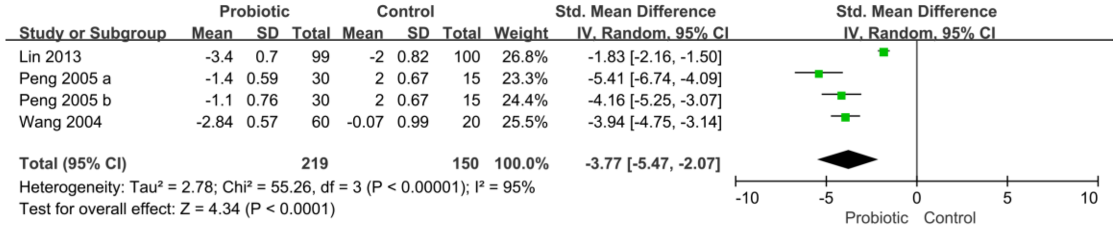

Supplementary Figure. 6 Forest plot for effect of probiotics on eye symptoms.

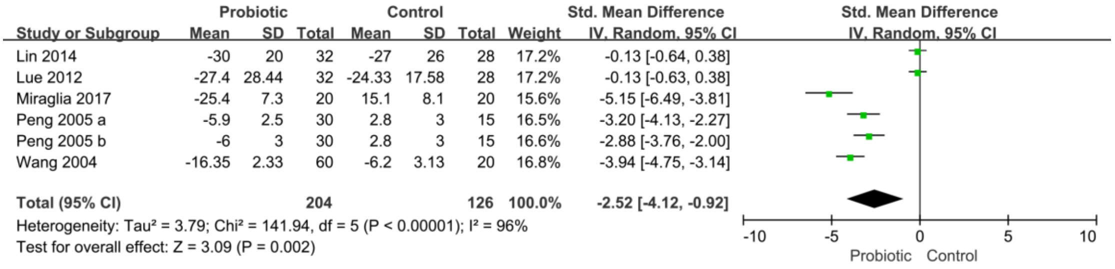

Supplementary Figure. 7 Forest plot for effect of probiotics on rhinoconjunctivitis quality of life questionnaire scores.

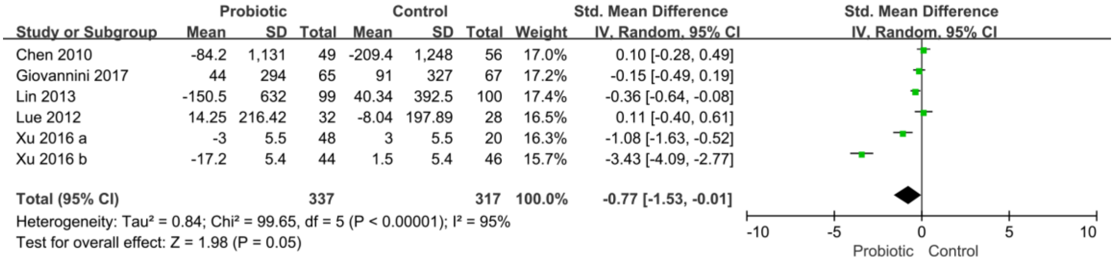

Supplementary Figure. 8 Forest plot for effect of probiotics on IgE.

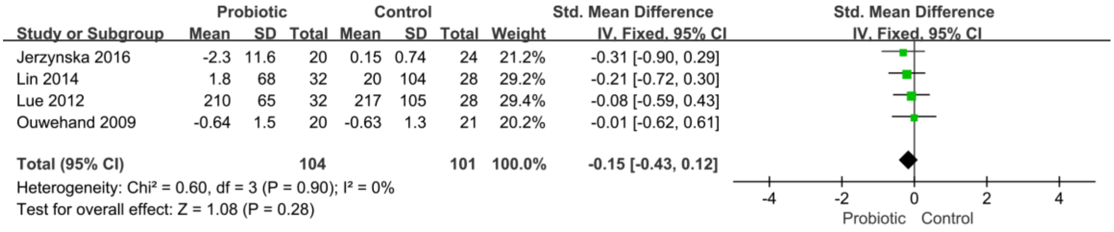

Supplementary Figure. 9 Forest plot for effect of probiotics on IL-10.

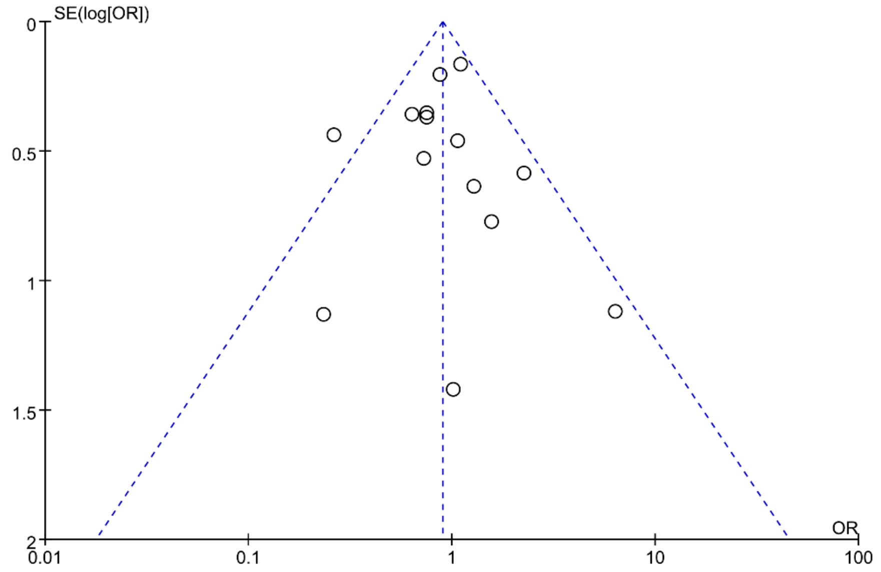

Supplementary Figure. 10 Funnel plot for the events of AR in children.
